# Idle medical students review emerging COVID-19 research

**DOI:** 10.1080/10872981.2020.1770562

**Published:** 2020-05-22

**Authors:** Carl Boodman, Santina Lee, Jared Bullard

**Affiliations:** a Section of Infectious Diseases, Department of Internal Medicine Max Rady College of Medicine, University of Manitoba, Winnipeg, Manitoba, Canada; b Department of Medical Microbiology and Infectious Diseases Max Rady College of Medicine University of Manitoba, Winnipeg, Manitoba, Canada; c Department of Pediatrics & Child Health Max Rady College of Medicine, University of Manitoba, Winnipeg, Manitoba, Canada; d Cadham Provincial Laboratory, Winnipeg, Manitoba, Canada

**Keywords:** COVID-19, interdisciplinary education, research, medical education, pandemic, school interruption

## Abstract

The coronavirus disease (COVID-19) pandemic is causing wide-spread interruptions in medical education. With little warning, clinical rotations were cancelled and medical students were sent home. While pre-clinical students transitioned to online curricula, clinical students were left without discreet educational goals. Simultaneously, medical doctors were scrambling to maintain competence in the face of rapidly evolving COVID-19 information. Here, we describe an education program that integrates medical students into interdisciplinary teams to review emerging COVID-19 research that directly answers questions sent in by medical doctors.

## Letter

In March 2020, clinical medical students at the University of Manitoba in Winnipeg, Canada were integrated into inter-professional teams to produce a weekly newsletter that directly responded to COVID-19 questions asked by doctors. Each team included librarians, graduate students and infectious disease fellows and was assigned to one of the following categories: clinical epidemiology, diagnosis, treatment, infection prevention and control and public health. A separate section focused on pediatric concerns. Questions were sent to a faculty lead who selected those that merited an urgent response. Four questions were assigned to each group and each medical student was assigned a question. Medical students with a particular interest could volunteer to answer a specific question. Librarians helped the medical students perform a literature review to ensure that all recent information was made available. During two weekly video-conferenced meetings, the quality, relevance and limitations of the articles were discussed. Medical students and graduate students then generated a formatted response to their question which was subsequently edited by the infectious disease fellows. A final review was performed by the staff lead prior to newsletter distribution and website uploading.

While many student groups produced summaries of COVID-19 articles, few incorporated structured inter-professional teams that addressed specific concerns voiced by medical doctors. On numerous occasions, medical leaders at the local, provincial and national level cited the newsletter as their most reliable weekly source of COVID-19 information. The newsletter was quickly distributed to thousands of healthcare providers nationally and internationally. Students recognized that their work influenced policy development and implementation. This augmented their engagement. The generation of the newsletter allowed students to implement numerous pedagogical skills. The students were able to practice research skills and perform literature searches. They then evaluated academic literature for limitations. Over the weeks, students expressed a deeper understanding of biases, confounding, intention-to-treat-analysis as well as the need for blinding, randomization and control. In a given week, a group of students became experts in their defined field, identifying conflicting information among publications. During the meetings and over email, students were able to perfect their inter-professional communication skills.

At first, certain students failed to meet deadlines, struggled to follow the response format and resisted the structured reference guide. This was addressed by one-on-one conversations with infectious disease fellows to allow for direct mentorship. Students were allowed to switch sections or drop-out of the project altogether. These techniques allowed elements of self-determination and contributed to sustainability. Overtime, progressively less involvement was required of the staff lead and fellows. This team-based model addressed discreet educational goals while contributing in a tangible way to an evolving pandemic and may have value outside of a pandemic.

